# Synthesis, Spectroscopic Characterization, and In Vitro Antimicrobial Studies of Pyridine-2-Carboxylic Acid N′-(4-Chloro-Benzoyl)-Hydrazide and Its Co(II), Ni(II), and Cu(II) Complexes

**DOI:** 10.1155/2012/104549

**Published:** 2012-10-22

**Authors:** Jagvir Singh, Prashant Singh

**Affiliations:** ^1^Department of Chemistry, Meerut College, Meerut 250001, India; ^2^Department of Applied Chemistry, B.B.A. University, Lucknow 226025, India

## Abstract

N-substituted pyridine hydrazide (pyridine-2-carbonyl chloride and 4-chloro-benzoic acid hydrazide) undergoes hydrazide formation of the iminic carbon nitrogen double bond through its reaction with cobalt(II), nickel(II), and copper(II) metal salts in ethanol which are reported and characterized based on elemental analyses, IR, solid reflectance, magnetic moment, molar conductance, and thermal analysis (TG). From the elemental analyses data, 1 : 2 metal complexes are formed having the general formulae [MCl_2_(HL)_2_] *·y*H_2_O (where M = Co(II), Ni(II), and Cu(II), *y* = 1–3). The important infrared (IR) spectral bands corresponding to the active groups in the ligand and the solid complexes under investigation were studied. IR spectra show that ligand is coordinated to the metal ions in a neutral bidentate manner with ON donor sites. The solid complexes have been synthesized and studied by thermogravimetric analysis. All the metal chelates are found to be nonelectrolytes. From the magnetic and solid reflectance spectra, the complexes (cobalt(II), nickel(II), and copper(II)) have octahedral and square planner geometry, respectively. The antibacterial and antifungal activity's data show that the metal complexes have a promising biological activity comparable with the parent ligand against bacterial and fungal species.

## 1. Introduction

**Scheme 1:**
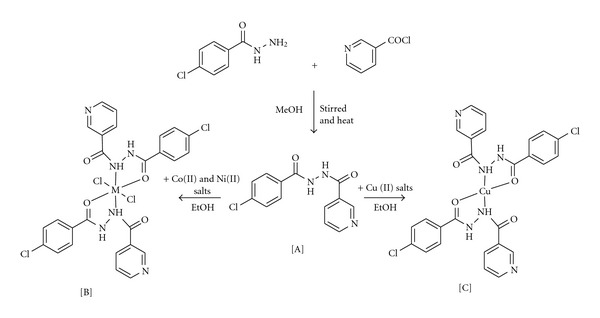
Synthesis of ligand (HL) and its metal complexes.

Hydrazones and their metal complexes have been given remarkable attention by the researchers, since they act as the most important stereochemical models in transition metal coordination chemistry, due to their preparative accessibility and structural variety. It has been suggested that the carbonyl linkage (C=O) in Hydrazide is responsible for their biological activities such as antitumor, antibacterial, antifungal, and herbicidal activities [1, 2]. Recently, the developments of new metal drug complexes have received special interests in the field of coordination chemistry [3, 4]. The transition metal-coordinated Hydrazones complexes play a significant role in many catalytic reactions like oxidations, asymmetric cyclopropanation, and polymerisation [5–7]. Hydrazide complexes of transition metal ions are known to provide useful models for elucidation of the mechanisms of enzyme inhibition by hydrazine derivatives [8] and for their possible pharmacological applications [9]. The activities of some such type complexes are very significant against Gram-positive bacteria in vitro. The derivatives of these chelate act as good potential oral drugs to treat the genetic disorders like thalassemia [10]. The structural characterization of these resultant hydrazide complexes revealed some interesting facts, such as their tendency and potency to act as planar pentadentate ligands in most of the complexes [11–14] along with tridentate character [15]. Moreover, these ligands exhibit ketoenol tautomerism and can coordinate in neutral [16], monoanionic [17], dianionic [4, 15, 16], or tetraanionic [18] forms, to the metal ions which have coordination numbers of six and seven [4, 18], generating mononuclear or binuclear species. However, it depends on the reaction conditions, such as metal ion, its concentration, the pH of the medium, and the nature of the hydrazide ligand [19]. Certain hydrazides and their Cu(II) complexes have antitumor activity. Transition metal complexes having the ability to bind and cleave DNA under physiological conditions are of current interests for their potential applications [20] in nucleic acids chemistry. Such complexes [21] are useful in foot printing studies, as sequence specific DNA binding agents, in genomic research and as diagnostic agents in medicinal applications. Much attention has been paid to biologically active metal complexes in recent years. Oxygen and nitrogen donor ligands [22] have been widely studied due to their high potential to coordinate with transition metals. Compounds containing carbonyl oxygen groups [23] have important position among organic reagents as potential donor ligands for the transition metal ions. Organic compounds and metal complexes [24] both display a wide range of pharmacological activity including anticancer, antibacterial, and fungi static effects. Though some reports are available on the hydrazones metal complexes [21, 22], there is no work focussed in the hydrazide derived from pyridine-2-carboxylic acid N′-(4-chloro-benzoyl)-hydrazide (HL) and its complexes (Scheme 1). In this paper, we report the synthesis, spectroscopic, thermal, and biological studies of the newly synthesized hydrazone complexes of the transition metal ions (Co(II), Ni(II), and Cu(II)).

## 2. Experimental

### 2.1. Materials

All the chemicals used in the present investigations were of the analytical reagent grade (AR). Pyridine-2-carbonyl chloride (BDH), 4-chloro-benzoic acid hydrazide (Sigma), metal salts, and solvents were purchased from Qualigens Chemicals Company, India. They were used as received. The elemental analysis (C, H, N) was performed using a Carlo-Erba 1106 Elemental Analyzer, and IR spectra were recorded on a Shimadzu-160 Spectrometer using KBr discs in the range 4000–400 cm^−1^. Electronic spectra were recorded on a Shimadzu-160 Spectrometer. The ^1^H and ^13^CNMR spectra were obtained on a Bruker DPX-400 Spectrometer using DMSO-d_6_ solvent and TMS as the internal reference at room temperature. The EPR spectra of the complexes were recorded as polycrystalline sample on a Varian E-4 EPR Spectrometer. The mass losses were measured in nitrogen atmosphere from ambient temperature up to 800°C at a heating rate of 10°C min^−1^. Molar conductivities in DMF or DMSO at 25°C were measured using a model CM-1 K-TOA company conductivity meter. Magnetic moments at 25°C were determined using the Gouy method with Hg [Co(SCN)_4_] as calibrant.

### 2.2. Synthesis of Hydrazine Ligand

In a round bottom flask (100 mL), a methanolic solution (10.0 mL) of pyridine-2-carbonyl chloride (0.02 m mol, 2.62 mL) and an aqueous methanolic solution (10 mL) of 4-chloro-benzoic acid hydrazide (0.01 m mol, 0.20 g) were taken and stirred at room temperature for 30 minutes after then the reaction was refluxed at 50°C for ~6 h. The resulting mixture was left under reflux for 3 h, and the formed solid product was separated by filtration, purified by crystallization from ethanol, washed with diethyl ether, and dried in a vacuum over anhydrous calcium chloride. The yellow product is produced in 52% yield.

### 2.3. Synthesis of Metal Complexes

The following detailed preparation is given as an example, and the other complexes were obtained similarly. The Cu(II) complex was synthesized by the addition of hot solution (60°C) of the Cu(II) chloride (0.17 g, 1 mmol) in an ethanol-water mixture (1 : 1, 25 mL) to the hot solution (60°C) of the ligand (0.550 g, 2 mmol) in the same solvent mixture (25 mL). The resulting mixture was stirred under reflux for one hour whereupon the complex precipitated. It was collected by filtration and washed with a 1 : 1 ethanol: water mixture and diethyl ether.

### 2.4. Analytical Data of Synthesized Ligand and Its Metal Complexes

#### 2.4.1. Ligand (HL)

Yield: 52%; M.P. 195°C, Mol. wt. 275, color: yellow; analytical data for (C_13_H_10_ClN_3_O_2_) found (calc.): C, 56.64 (56.11); H, 3.66 (3.11); N, 15.24 (14.97). IR (KBr, cm^−1^) 3440 *ν*_NH_, 1690 *ν*_C=O_, 3015 *ν*_C–H_, 2228 *ν*_C–N_. ESI-MS, m/z Data found (calc.): 276 (275), ^1^H NMR (DMSO-d_6_) *δ* ppm: 7.1 (m, 8H, HC-Ar), 3.8 (s, 2H, NH–NH). ^13^C NMR (DMSO-d_6_) *δ* ppm: 117.53–121.07 (10C, CH-Ar.), 143.23 (1C, C–N), 153.88 (2C, C=O).

#### 2.4.2. Complex I

Yield: 65%; MP: 230°C; Mol. wt. 681; color: reddish; analytical data for [C_26_H_20_Cl_4_CoN_6_O_4_] found (calc.): C, 45.84 (31.15); H, 2.96 (2.85); N, 12.34 (12.17); (KBr, cm^−1^) 3406 *ν*_NH_, 1690 *ν*_C=O_, 3015 *ν*_C–H_, 2203 *ν*_C–N_, 519 *ν*_M–N_, 416 *ν*_M–O_. ^1^H NMR (DMSO-d_6_) *δ* ppm: 7.1 (m, 8H, HC-Ar), 3.01 (s, 2H, NH–NH). ^13^C NMR (DMSO-d_6_) *δ* ppm: 117.53–121.07 (10C, CH-Ar.), 133.22 (1C, C–N), 153.88 (2C, C=O).

#### 2.4.3. Complex II

Yield: 42%; MP: 235°C; Mol. wt. 680; color: greenish; analytical data for [C_26_H_20_Cl_4_N_6_NiO_4_] found (calc.): C, 45.86 (45.55); H, 2.96 (2.55); N, 12.34 (12.12). (KBr, cm^−1^) 3400 *ν*_NH_, 1690 *ν*_C=O_, 3015 *ν*_C–H_, 2210 *ν*_C–N_, 518 *ν*_M–N_, 408 *ν*_M–O_. ^1^H NMR (DMSO-d_6_) *δ* ppm: 7.1 (m, 8H, HC-Ar), 3.5 (s, 2H, NH–NH). ^13^C NMR (DMSO-d_6_) *δ* ppm: 117.53–121.07 (10C, CH-Ar.), 136.30 (1C, C–N), 153.88 (2C, C=O).

#### 2.4.4. Complex III

Yield: 48%; MP: 220°C; Mol. wt. 615; color: brownish; analytical data for [C_26_H_20_Cl_2_CuN_6_O_4_] found (calc.): C, 50.78 (50.30), H, 3.28 (2.90), N, 13.67 (12.17). (KBr, cm^−1^) 3400 *ν*_NH_, 1690 *ν*_C=O_, 3015 *ν*_C–H_, 2200 *ν*_C–N_, 512 *ν*_M–N_, 420 *ν*_M–O_. ^1^H NMR (DMSO-d_6_) *δ* ppm: 7.1 (m, 8H, HC-Ar), 3.4 (s, 2H, NH–NH). ^13^C NMR (DMSO-d_6_) *δ* ppm: 117.53–121.07 (10C, CH-Ar.), 131.20 (1C, C–N), 153.88 (2C, C=O).

### 2.5. Biological Activity

Antimicrobial activity of the tested samples was determined using a modified Kirby-Bauer disc diffusion method [25]. One hundred microliters of the tested bacteria or fungi were grown in 10 mL of fresh media until they reached account of approximately 98 cells/mL for bacteria and 95 cells/mL for fungi [26]. One hundred microliters of microbial suspension was spread onto agar plates corresponding to the broth in which they were maintained. Isolated colonies of each organism that might be playing a pathogenic role should be selected from primary agar plates and tested for susceptibility by disc diffusion method [27]. Of the many media available, NCCLS recommends Mueller-Hinton agar due to its results in good batch-to-batch reproducibility. Disc diffusion method for filamentous fungi tested by using approved standard method (M38-A) developed [28]. For evaluating the susceptibilities of filamentous fungi to antifungal agent, the disc diffusion method was applied for yeast developed by using approved standard method (M44-P) [29]. Plates inoculated with filamentous fungi as *C. albican* MTCC 227 at 25°C for 48 h; Gram (+) bacteria as *Staphylococcus aureus* MTCC 3160; Gram (−) bacteria as *Staphylococcus aureus* MTCC 25923, they were incubated at 35–37°C for 24–28 h and yeast as *S. cerevisiae* MTCC 361 incubated at 30°C for 24–28 h and, and then the diameters of the inhibition zones were measured in millimeters [29]. Standard discs of Gentamycin (antibacterial agent) and amphotericin-B (antifungal agent) served as positive controls for antimicrobial activity, but filter discs impregnated with 10 ML of solvent (distilled water, chloroform, DMSO) were used as a negative control. The agar used is Mueller-Hinton agar that is rigorously tested for composition and pH. Further the depth of the agar in the plate is a factor to be considered in the disc diffusion method. This method is well documented, and standard zones of inhibition have been determined for susceptible and resistant values. Blank paper discs (Schleicher and Schuell, Spain) with a diameter of 8.0 mm were impregnated with 10 ML of tested concentration of the stock solutions. When a filter paper disc impregnated with a tested chemical is placed on agar, the chemical will diffuse from the disc into the agar. This diffusion will place the chemical in the agar only around the disc. The solubility of the chemical and its molecular size will determine the size of the area of chemical infiltration around the disc. If an organism is placed on the agar, it will not grow in the area around the disc if it is susceptible to the chemical. This area of no growth around the disc is known as zone of inhibition or clear zone. For the disc diffusion, the zone diameters were measured with slipping calipers of the national committee for clinical laboratory standards [29]. Agar-based methods such Etest and disc diffusion can be good alternatives because they are simpler and faster than broth-based methods [30].

## 3. Result and Discussion

The new prepared hydrazide ligand is subjected to elemental analyses, IR, Mass bar and ^1^H & ^13^C NMR spectral studies. The results obtained are in good agreement with those calculated for the suggested formula, and the melting point is sharp indicating the purity of the prepared ligand (HL). The structure of HL under study is given in Scheme 1. The IR spectra of hydrazide ligand contain a strong C=O absorption band at 1670 cm^−1^and N–H absorption band at 3187 cm^−1^, and after complexation, these bands are slightly shifted to lower wave numbers (1646–1649 cm^−1^ and 3133–3140 cm^−1^) indicating the involvement of the oxygen and nitrogen atom in chelate formation, respectively. In the FT IR spectra of all the complexes, the nonligand bands observed at 520–586 and 420–466 cm^−1^ regions can be assigned to (M–O) and (M–N), respectively [31–33]. ^1^H NMR spectrums showed signals in the range *δ* 8.03 ppm, and these signals were the evidence of the secondary amide bonding to the ligand [34]. ^13^C NMR spectrum of complexes displays a signal at *δ* 167.45 ppm and *δ* 163.54 ppm, due to (C–NH) and (C=O), which was indicating that carbon atoms of carbon-amide group and oxygen atom of carbonyl group participate the formation of ligand [28]. The spectrum shows the molecular ion peak at m/z = 275 (C_13_H_10_ClN_3_O_2_, calculated atomic mass 276 amu due to ^13^C and ^15^N isotopes). The different competitive fragmentation pathways of ligand give the peaks at different mass numbers at 304. The intensity of these peaks reflects the stability and abundance of the ions. The presence of fragments at m/z values 291, 276, 249, and 237 shows the fragmentation. The mass spectrum clearly suggests existence of ligand in the hydrazine form. The molar conductance values of all the complexes lie in the range 122–165 ohm^−1 ^cm^2^ mole^−1^ corresponding to 1 : 2 nonelectrolytic behaviors [29]. The electronic spectra of the cobalt(II) complex showed three bands at 8780–8810, 17475–17775 and 30235–30270 cm^−1^, which may be assigned to ^4^T_1g_ → ^4^T_2g_ (F), ^4^T_1g_ → ^4^T_1g_ (P), and ^4^T_1g_ → ^3^A_2g_ (F) transitions and suggested octahedral geometry around the cobalt ion. The magnetic moment 4.80–488 BM is an addition evidence for an octahedral structure [35].

**Figure 1:**
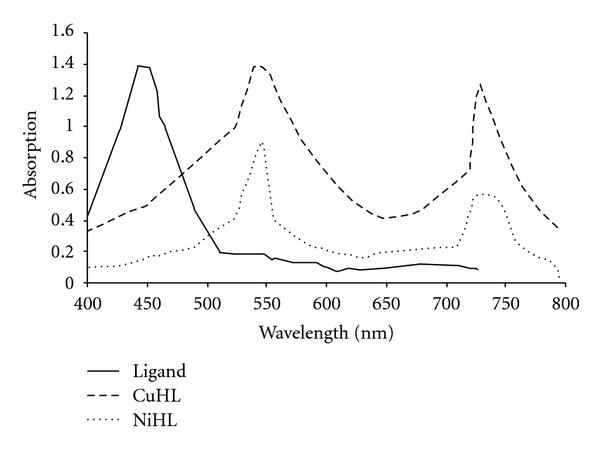
UV-visible spectra of free ligand, Cu-complex, and Ni-complex.

**Figure 2:**
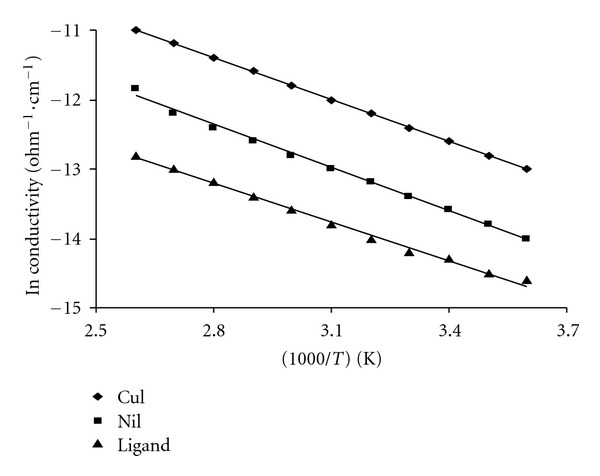
The DC conductivities of free ligand, Cu-complex, and Ni-complex before doping.

**Figure 3:**
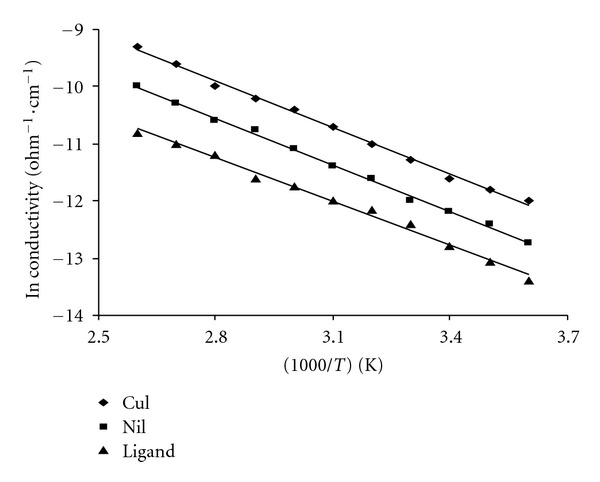
The DC conductivity of free ligand, Cu-complex, and Ni-complex after doping.

The cobalt complexes showed magnetic moment values 4.70–490 B.M. at room temperature. These high values of the magnetic moments and the stoichiometries suggest a coordination number of six for the central cobalt ions and an octahedral geometry. The magnetic properties of copper complex may be divided into broad classes. First, those are having essentially temperature-independent magnetic moments in rang 2.20 BM. Those exhibiting such moments are mononuclear complexes having no major interaction between the unpaired electrons on different copper ion. The moments in such complex, as in apparent, lie appreciably above the spine-only value (1.73 BM), but as the electronic ground states are nondegenerate, this cannot arise from inherent angular momentum in the ground state. It arises due to mixing of some orbital angular momentum from excited states via spin orbit coupling. The copper (II) complexes exhibit magnetic moments of 1.70–1.75 B.M., respectively, at room temperature. The electronic spectra of the copper(II) complexes display a broad band at 14220–14918 cm^−1^ due to ^2^B_1g_ → ^2^E_g_ and two bands at 16390–16550 and 27250–27350 cm^−1^ assigned to d-d transitions and a charge transfer band, respectively, of square planner environment [31]. The observed magnetic moment of the copper complexes is 1.75–180 BM. The Ni(II) complex is found to have a room temperature magnetic moment value of 3.87 B.M., which is in the normal range observed for octahedral Ni(II) complexes [26, 34, 36]. The electronic spectrum displays three bands in the solid reflectance spectrum at m_1_: 14968 cm^−1^; ^3^A_2g_→ ^3^T_2g_; m_2_: 17,788 cm^−1^; ^3^A_2g_ → ^3^T_1 g_ (F) and m_3_; 21347 cm^−1^; ^3^A_2g_ → ^3^T_1g_ (P). The spectrum shows also a band at 24565 cm^−1^ which may be attributed to ligand to metal charge transfer (Figure 1). The maximum conductivity value was 1.81 × 10^−4^ ohm^−1 ^cm^−1^ for doped copper(II) complex because of the small size of nickel atom compared with iron atom. Also the prepared hydrazide complexes at different temperatures (303–373) K show that the increased conductivity with increasing of temperatures may be attributed to presence of metals (d-d*) transition. Figures 2 and 3 show that the conductivities of doped and undoped compounds increase with increasing of temperatures which is consistent with semiconductors properties [19, 20].

The dopping compounds have higher conductivities than undoped because iodine doping leads to oxidation of iodine molecules to form I_3_^−1^, I_5_^−3^, and reduction of hydrazide complexes molecules, this effect increase the conductivity by making acceptor bands, and the distance between the energy levels was low. The DC electrical conductivities of doped and undoped prepared compounds over temperature range (0–110°C) and under vacuum were measured by using conductivity apparatus which is consisting of temperature recorder, power supply, voltmeter, resistance and sample cell. The studied samples were as discs covered in two sides by silver paint. The ligand, Cu(II), and Ni(II) complexes were doped with iodine by mixing 1 g (0.004 mole) ligand, 1 g (0.0022 mole) Cu-complex, and 1 g (0.002 mole) Ni-complex with 25 mL of iodine solution in CCL_4_ (4%, w/v), the mixture was refluxed with stirring for 48 hours and then filtered and dried in the vacuum oven at 50°C. The conductivities at different temperatures were calculated according to Arrhenius equation as shown below [19]:

(1) $$\begin{array}{c} \sigma =\sigma \text{o}*\exp\left(-\frac{\Delta E}{2K}\right). \end{array}$$

The resistance of the sample and its electrical conductivity is calculated from the equations:

(2) $$\begin{array}{c} R_{x}=\frac{R_{s}*V_{x}}{V_{s}},\;\;\sigma \;=\;\left(\frac{L}{A}\right)*\frac{1}{R_{x}}, \end{array}$$

where *R*_*s*_ is standard resistance (ohm), *R*_*x*_ is sample resistance (ohm), *V*_*s*_ is standard Voltage (volt),*V*_*x*_ is sample voltage (volt), *L* is Sample thickness (cm), and *A* is The painted area of the sample surface (cm^2^).

### 3.1. Thermal Analysis (TG)

The thermodynamic activation parameters of decomposition processes of dehydrated complexes, namely, activation energy, enthalpy, entropy, and Gibbs free energy change of the decomposition are evaluated graphically by employing the Coats-Redfern relation. The entropy of activation, enthalpy of activation, and the free energy change of activation were calculated. The high values of the activation energies reflect the thermal stability of the complexes. The entropy of activation is found to have negative values in all the complexes which indicate that the decomposition reactions proceed spontaneously. The thermogram of (CoCl_2_(HL)) 3H_2_O and (CuCl_2_(HL)) 2H_2_O chelates show five decomposition steps within the temperature range 30–900 and 50–950°C, respectively. The first two steps of decomposition within the temperature range 30–250 and 50–230°C correspond to the loss of three water molecules of hydration, Cl_2_, one coordinated water molecule, and loss of two water molecules of hydration and coordination and Cl_2_ (in case of Cu(II) complex, mass loss of 29.37% (calcd. 28.57%)). The energy of activation was found to be 44.40 and 78.24 (in case of Co(II) complex) and 36.52 and 53.78 kJ mol^−1^ (in case of Cu(II) complex) for the first and second steps, respectively. The subsequent steps (230–950°C) correspond to the removal of the organic part of the ligand leaving metal oxide as a residue. The overall weight loss amounts to 86.02% (calcd. 85.51%) and 84.26 (calcd. 84.11%) for Co(II) and Cu(II) complexes, respectively. The TG curves of the Ni(II) chelate show four stages of decomposition within the temperature range of 30–950°C. The first step at 30–130°C corresponds to the loss of water molecules of hydration. The subsequent three steps (2nd, 3rd, and 4th) involve the loss of Cl_2_ and ligand molecule. The overall weight loss amounts to 84.97% (calcd. 85.42%), and the activation energy is 102.4–243.4 Ni(II) chelates.

### 3.2. Biological Activity

In testing the antibacterial activity of these compounds we used more than one test organism to increase the chance of detecting antibiotic principles in tested materials. The sensitivity of a microorganism to antibiotics and other antimicrobial agents was determined by the assay plates which incubated at 28°C for 2 days for yeasts and at 37°C for 1 day for bacteria. All of the tested compounds showed a remarkable biological activity against different types of Gram-positive and Gram-negative bacteria and against fungi species. The data are listed in Table 1.

It was demonstrated that the newly prepared ligand and its metal complexes showed a higher effect on *C. albican* (Gram-negative bacteria) and *S. aureus* (Gram-positive bacteria). It is known that the membrane of Gram-negative bacteria is surrounded by an outer membrane containing lipopolysaccharides. The newly synthesized hydrazide and its metal complexes seem to be able to combine with the lipophilic layer in order to enhance the membrane permeability of the Gram-negative bacteria. The lipid membrane surrounding the cell favours the passage of only lipid soluble materials; thus the lipophilicity is an important factor that controls the antimicrobial activity. Also the increase in lipophilicity enhances the penetration of hydrazide and its metal complexes into the lipid membranes and thus restricts further growth of the organism. This could be explained by the charge transfer interaction between the studied molecules and the lipopolysaccharide molecules which lead to the loss of permeability barrier activity of the membrane. The hydrazide and its metal complexes could enhance the antimicrobial effect on both strains probably by the hydroxyl group. The Co(II), Ni(II), and Cu(II) complexes were almost the most promising broad spectrum antimicrobial agents due to the presence of coordinated anion with higher antimicrobial activity than the other complexes. From the data the inhibition zone of the metal chelates is higher than that of the ligand. Such increased activity of the metal chelates is due to the lipophilic nature of the metal ion in complexes. Furthermore, the mode of action of hydrazide of the compounds may involve the formation of a hydrogen bond through the azomethine nitrogen atom with the active canters of all the constituents, resulting in interference with normal cell process. 

## 4. Conclusion

The results of this investigation support the suggested structures of the metal complexes. It is obvious from this study that only mononuclear complexes are obtained. The IR spectral studies reveal that HL coordinated to the metal ions via C=O and amide NH–NH group. The chelates are nonelectrolytes. All metal cations have octahedral geometry except copper (II) chelate. The thermal decomposition of the complexes as well as the thermodynamic parameters is studied. The biological activities of the hydrazide under investigation and its complexes against bacterial and fungal organisms are promising which need further and deep studies on animals and humans.

## Figures and Tables

**Table 1:** Antimicrobial activity of ligand and their metal complexes.

Compounds	Time hrs	*S. aureus* MTCC 3160		*S. aureus* MTCC 25923		*C. albican* MTCC 227		*S. cerevisiae* MTCC 361	
		Diameter of zone of inhibition (mm) 100 *μ*g 50 *μ*g		Diameter of zone of inhibition (mm) 100 *μ*g 50 *μ*g		Diameter of zone of inhibition (mm) 100 *μ*g 50 *μ*g		Diameter of zone of inhibition (mm) 100 *μ*g 50 *μ*g	
HL	24	10	11	—	—	—	—	13	15
	48	12	15	—	—	—	—	13	16
[1]	24	10	12	13	16	19	17	19	14
	48	11	12	14	19	11	10	12	17
[2]	24	14	13	16	18	15	27	21	18
	48	—	—	—	—	11	29	12	19
[3]	24	—	—	—	—	13	10	12	10
	48	14	10	13	12	14	13	14	11
Gentamycin	24	22	24	22	24	—	—	—	—
	48	22	24	22	24	—	—	—	—
Amphotericin-B	24	—	—	—	—	17	21	17	21
	48	—	—	—	—	17	21	17	21

## References

[1] Chohan ZH, Praveen M, Ghaffar A (1997). Structural and biological behaviour of Co(II), Cu(II) and Ni(II) metal complexes of some amino acid derived Schiff-bases. *Metal-Based Drugs*.

[2] Chohan ZH, Kausar S (1993). Synthesis, structural and biological studies of nickel(II), copper(II) and zinc(II) chelates with tridentate Schiff bases having NNO and NNS donor systems. *Chemical and Pharmaceutical Bulletin*.

[3] Seven MJ, Johnson LA (1960). *Metal Binding in Medicine*.

[4] Srivastava RS (1997). Studies on some antifungal transition metal chelates of 2-(2-hydroxybenzylideneamino) benzimidazole. *Indian Journal of Chemistry*.

[5] Patel VK, Vasanwala AM, Jejurkar CN (1976). Synthesis of mixed Schiff base complexes of copper(II) and nickel(II) and their spectral, magnetic and antifungal studies. *Indian Journal of Chemistry*.

[6] Maggio F, Pellerito A, Pellerito L, Grimaudo S, Mansueto C, Vitturi R (1994). Organometallic complexes with biological molecules—II. Synthesis, solid-state characterization and in vivo cytotoxicity of diorganotin(IV)chloro and triorganotin(IV) chloro derivatives of penicillin G. *Applied Organometallic Chemistry*.

[7] Vitturi R, Mansueto C, Gianguzza A, Maggio F, Pellerito A, Pellerito L (1994). Organometallic complexes with biological molecules—III: in vivo cytotoxicity of diorganotin(IV)chloro and triorganotin(IV)chloro derivatives of penicillin g on chromosomes of *Aphanius fasciatus*(pisces, cyprinodontiformes). *Applied Organometallic Chemistry*.

[8] Pellerito L, Maggio F, Consigilo A, Pellerito A, Stocco GC, Gremaudo S (1995). Organometallic complexes with biological molecules—4: di- and tri-organotin(IV)amoxicillin derivatives: solid-state and solution-phase spectroscopic investigations. *Applied Organometallic Chemistry*.

[9] Vitturi R, Zava B, Colomba MS, Pellerito A, Maggio F, Pellerito L (1995). Organometallic complexes with biological molecules—5: in vivo cytotoxicity of diorganotin(IV)-amoxicillin derivatives in mitotic chromosomes of *Rutilus rubilio*(pisces, Cyprinidae). *Applied Organometallic Chemistry*.

[10] Rosenberg B, VanCamp L (1970). The successful regression of large solid sarcoma 180 tumors by platinum compounds. *Cancer Research*.

[11] Cleare MJ, Hoeschele JD (1973). Studies on the antitumor activity of group VIII transition metal complexes—I. Platinum (II) complexes. *Bioinorganic Chemistry*.

[12] Rosu T, Pahontu E, Reka-Stefana M (2012). Synthesis, structural and spectral studies of Cu(II) and V(IV) complexes of a novel Schiff base derived from pyridoxal and antimicrobial activity. *Polyhedron*.

[13] Crowe AJ (1988). The antitumour activity of tin compounds. *Metal Based Antitumour Drugs*.

[14] Williams DR (1972). Thermodynamic considerations in co-ordination—part 10. a potentiometric and calorimetric investigation of copper(II) histidine complexes in solution. *Journal of the Chemical Society, Dalton Transactions*.

[15] Sakiyan I, Loğoğlu E, Arslan S, Sari N, Şakiyan N (2004). Antimicrobial activities of N-(2-hydroxy-1-naphthalidene)-amino acid(glycine, alanine, phenylalanine, histidine, tryptophane) Schiff bases and their manganese(III) complexes. *BioMetals*.

[16] Sari N, Arslan S, Logoglu E, Sakiyan I (2003). Antibacterial activities of some Amino acid Schiff bases. *Journal of Animal Science*.

[17] Williams RJP (1968). Role of transition metal ions in biological processes. *Royal Institute of Chemistry, Reviews*.

[18] Maurya RC, Mishra DD, Pillai S (1997). Studies on some mixed-ligand chelates of cobalt(II) involving acetoacetylarylamides and biologically active heterocyclic oxygen donors. *Synthesis and Reactivity in Inorganic and Metal-Organic Chemistry*.

[19] Maurya RC, Verma R, Trivedi PK, Singh H (1998). Synthesis, magnetic and spectral studies of some mixed-ligand chelates of Bis(2-hydroxyacetophenonato)copper(II) with 2-or 3-pyrazoline-5-one derivatives. *Synthesis and Reactivity in Inorganic and Metal-Organic Chemistry*.

[20] Singh RB, Jain P, Singh RP (1982). Hydrazones as analytical reagents: a review. *Talanta*.

[21] Tan C, Liu J, Chen L, Shi S, Ji L (2008). Synthesis, structural characteristics, DNA binding properties and cytotoxicity studies of a series of Ru(III) complexes. *Journal of Inorganic Biochemistry*.

[22] Zuber G, Quada JC, Hecht SM (1998). Sequence selective cleavage of a DNA octanucleotide by chlorinated bithiazoles and bleomycins. *Journal of the American Chemical Society*.

[23] Hecht SM (2000). Bleomycin: new perspectives on the mechanism of action. *Journal of Natural Products*.

[24] Ghosh T, Maiya BG, Samanta A (2005). Mixed-ligand complexes of ruthenium(II) containing new photoactive or electroactive ligands: synthesis, spectral characterization and DNA interactions. *Journal of Biological Inorganic Chemistry*.

[25] Carcelli M, Pelizzi C, Pelizzi G, Mazza P, Zani F (1995). The different behaviour of the di-2-pyridylketone 2-thenoylhydrazone in two organotin compounds. Synthesis, X-ray structure and biological activity. *Journal of Organometallic Chemistry*.

[26] Singh NP, Anu, Singh JV (2012). Magnetic and spectroscopic studies of the synthesized metal complexes of bis(pyridine-2-carbo) hydrazide and their antimicrobial studies. *E-Journal of Chemistry*.

[27] Saha S, Dhanasekaran D, Chandraleka S, Thajuddin N, Panneerselvam A (2010). Synthesis, characterization and antimicrobial activity of cobalt metal complexes against drug resistant bacterial and fungal pathogens. *Advances in Biological Research*.

[28] Mulligan ME, Murray-Leisure KA, Ribner BS (1993). Methicillin-resistant *Staphylococcus aureus*: a consensus review of the microbiology, pathogenesis, and epidemiology with implications for prevention and management. *American Journal of Medicine*.

[29] Farrell N (2003). Metal complexes as drugs and chemotherapeutic agents. *Comprehensive Coordination Chemistry*.

[30] Dholakiya PP, Patel MN (2004). Metal complexes: preparation, magnetic, spectral, and biocidal studies of some mixed-ligand complexes with schiff bases containing NO and NN donor atoms. *Synthesis and Reactivity in Inorganic and Metal-Organic Chemistry*.

[31] Rehman SU, Chohan ZH, Gulnaz F, Supuran CT (2005). In-vitro antibacterial, antifungal and cytotoxic activities of some coumarins and their metal complexes. *Journal of Enzyme Inhibition and Medicinal Chemistry*.

[32] Lee U, Koo BK (2005). Synthesis and crystal structures of Mn(II), Co(II), Ni(II), Cu(II), and Zn(II) metal complexes with NNO functionalized ligands. *Bulletin of the Korean Chemical Society*.

[33] Valdés-Martínez J, Toscano RA, Zentella-Dehesa A, Salberg MM, Bain GA, West DX (1996). Synthesis, crystal and molecular structure of 5-bromo-salicylaldehyde-2-methylthiosemicarbazonato(nitrato)copper(II) monohydrate. *Polyhedron*.

[34] Geary WJ (1971). The use of conductivity measurements in organic solvents for the characterisation of coordination compounds. *Coordination Chemistry Reviews*.

[35] Cotton FA, Wilkinson G, Murillo CA, Bochmann M *Advanced Inorganic Chemistry*.

[36] Vogel AI (1978). *A Textbook of Quantitative Inorganic Analysis*.

